# Furin Expression in Patients With Psoriasis—A Patient Cohort Endangered to SARS-COV2?

**DOI:** 10.3389/fmed.2021.624462

**Published:** 2021-02-10

**Authors:** Thomas Graier, Nicole Golob-Schwarzl, Wolfgang Weger, Theresa Benezeder, Clemens Painsi, Wolfgang Salmhofer, Peter Wolf

**Affiliations:** ^1^Department of Dermatology and Venereology, Medical University of Graz, Graz, Austria; ^2^Department of Dermatology and Venereology, State Hospital, Klagenfurt, Austria

**Keywords:** psoriasis, psoriatic arthritis, secukinumab, ustekinumab, COVID-19, SARS-CoV-2, furin, angiotensin converting enzyme-2

## Abstract

**Background:** SARS-Cov2 has raised concerns among dermatologists regarding psoriasis and its respective treatments. Comorbidities, which induce the expression of the proprotease furin have been associated with severe course of COVID-19. Furin and angiotensin converting enzyme 2 (ACE2) play a major role in viral host cell entry of SARS-Cov2.

**Objective:** To evaluate mRNA expression of Furin and ACE2 from blood cells in psoriasis patients, and whether systemic or topical treatment reduces expression levels.

**Methods:** This observational translational study analyzed blood samples from patients from a clinical trial and samples retrieved from the biobank of the Psoriasis Registry Austria (PsoRA). Furin and ACE2 expression levels were analyzed prior to as well as 3 and 12–24 months after start of biologic treatment with either ustekinumab or secukinumab. Additionally, the study analyzed expression levels prior to, 6 days after start of dithranol treatment and 4–6 weeks after end of dithranol treatment.

**Results:** Furin mRNA expression was significantly increased at baseline in the biologic (4.9 ± 2.6 fold, *p* < 0.0001) and in the dithranol group (2.7 ± 1.4 fold, *p* < 0.001) compared to controls. There was a trend for arthritis patients to express more furin than patients with psoriatic skin involvement only (5.26 ± 2.30 vs. 3.48 ± 2.27, *p* = 0.078). Analyzing furin mRNA expression after treatment initiation with secukinumab or ustekinumab revealed a normalization of levels after 3 and 12 to 24 months. Similar findings were obtained for patients treated with dithranol, with significantly decreased expression levels 6 days after start of dithranol treatment and also at follow-up, (4–6 weeks after dithranol treatment had been terminated). ACE2 expression levels did not differ from controls at any timepoint, regardless of biologic or topical treatment.

**Conclusion:** Significantly overexpressed levels of furin were observed in untreated patients, and, thus, these patients may be at risk for infection and a severe course of COVID-19. However, the data indicate that successful therapeutic intervention in psoriasis, by systemic biologic or topical treatment, can efficiently reduce furin levels in blood cells, possibly limiting the risk of psoriasis patients for a severe COVID-19 course.

**Clinical Trial Registration:** ClinicalTrials.gov, identifier NCT02752672.

## Introduction

The recent outbreak of SARS-CoV2 has raised concerns among dermatologists regarding psoriasis and its respective treatments, especially concerning biologic treatment (1–3), as the condition itself and its treatments bear the risk for infections (4, 5), ultimately leading to considerations, whether or not biologic or conventional treatment should be interrupted during the ongoing pandemic (3, 6–8). First reports from observational studies have not found evidence for an increased hospitalization rate or mortality for patients with psoriasis under either conventional systemic or biologic treatment (9–12). Similar findings have been observed for patients with rheumatological diseases; in fact, lower odds rates of hospitalization have been observed in patients treated with TNF-α- inhibitors (13). However, it remains unknown, whether or not a rheumatological condition *per se* increases the risk for a severe course of COVID-19 infection (13). At present, factors associated with increased COVID-19 mortality and hospitalization rate are hypertension, diabetes and coronary heart disease (14), which also accounts for patients with rheumatological conditions with metabolic or cardiovascular comorbidities (13). A plausible link between patients‘ comorbidities and COVID-19 severity has been recently suggested from physiologists (15, 16), pointing to furin as predictor for disease severity.

The proprotein convertase furin is an ubiquitously expressed protein and can be found in all mammalian tissues. It belongs to the subtilisin/kexin family (PCSK) that play a pivotal role in several physiological as well as pathophysiological processes. High plasma furin concentrations are found in patients with dysmetabolic phenotypes and diabetes (17, 18) where furin is critically involved and upregulated in atherosclerotic plaque formation (19) as well as in heart failure (20). Furthermore, it is upregulated in activated immune cells. Although its role is not fully understood to date, it is considered to activate anti-inflammatory cytokines (TGF-β1) and thus, directly modulates CD4+ and CD8+ cell activity (21–24). Furthermore, furin is also upregulated in blood T-cells from patients with rheumatoid arthritis (RA) (25, 26), systemic lupus erythematosus (27) and primary Sjögren syndrome (28).

Furin plays a crucial role in processing the viral surface glycoproteins (29), making its inhibitors promising drugs for the treatment of infectious diseases (30, 31). It has recently gained attention, as SARS-Cov2 appears to harbor a cleavage site for furin-like proteases, that facilitate the binding of fusion domains, which are essential for the entry of the virus into the cell (15, 16, 32–34). Furin conceivably exerts its action intracellularly, as well as extracellularly, by circulating in the blood, though to a lesser extend (35). It is upregulated in T-cells, which are activated during infections, and circulate through the body (36). Another virus using furin in its cell entry mechanism is the Middle East Respiratory Syndrome related coronavirus (MERS-CoV), for which the efficient invasion of CD4+ and CD8+ T-cells (driving them into apoptosis) from peripheral blood as well as lymphoid organs during infections has already been described (37). Additionally, reduced T-cell counts and functional exhaustion of remaining T-cells has also been described for SARS-Cov2 (38) and lymphopenia serves as a prognostic factor for worse outcome (37–40). Therefore, the increased availability of furin in dysmetabolic states may induce a furin-facilitated coronavirus replication, that may be responsible for hypersensitive immunological response (cytokine storm) in some patients, as recently suggested (16).

Another protein, that is, carefully monitored by scientists is Angiotensin Coverting Enzyme 2 (ACE2), which mainly exerts its function in the lung (34, 41). SARS-Cov2 binds to ACE2 with higher affinity than other coronaviruses and is considered as essential mechanism for viral entry in human cells (42). However, since ACE2 is commonly targeted in antihypertensive treatment as well, it has been hypothesized that those patients may also have an increased risk for severe infection, due to an upregulated ACE2 expression caused by its inhibitors (43). However, antihypertensive treatment has not been associated with respiratory distress or mortality in hospitalized patients, but it remains unknown if it increases the risk for hospitalization (44).

In order to identify a potentially increased vulnerability of psoriasis patients to COVID-19, we analyzed furin and ACE2 mRNA levels from blood cells in psoriasis patients prior to and during systemic biologic treatment or prior to and after topical dithranol treatment.

## Methods

### Patient and Sample Characteristics

Blood samples were available from two patient groups, that is, from a study on topical dithranol in psoriasis (45) and from the cohort of psoriasis patients of the Psoriasis Registry Austria (PsoRA). Inclusion criteria of the dithranol study were diagnosis of chronic plaque psoriasis, and age above 18 years. Exclusion criteria were intolerance of dithranol, autoimmune diseases, general poor health status, pregnancy and breast-feeding, topical treatment (steroids, vitamin D3-analogs and/or Vitamin A acid-derivates) within 2 weeks, and phototherapy within 4 weeks prior to study enrollment. In total, 15 psoriasis patients (11 men, 4 women) were enrolled in the dithranol study (45). Blood from 17 patients (13 men, 4 women) with moderate to severe chronic psoriasis [enrolled in the Psoriasis Registry Austria (PsoRA)] was available for the analysis with regard to treatment under daily life conditions with biologics (from 12 patients treated with secukinumab and 5 patients treated with ustekinumab). Blood samples of patients undergoing surgery for removal of benign skin lesions were available from 12 subjects for control purposes. The samples were from patients who did not suffer from psoriasis, other inflammatory diseases or autoimmune diseases.

### Blood Sampling

Blood samples were taken in the dithranol study from psoriasis patients before (baseline), early during treatment at first strong perilesional inflammation around day 6 and at a follow-up visit (4–6 weeks after end of therapy). Dithranol ointment was prepared and administered, as previously described (45). Blood samples for the biologic study were taken from psoriasis patients at baseline (before start of treatment), after 3 months, and after 12–24 months therapy with ustekinumab and secukinumab. The blood samples were stored at −80°C until RNA extraction for analysis.

### RNA Extraction

RNA was extracted from frozen PAXgene blood RNA tube of psoriasis patients and control subjects. Total RNA was extracted using the PAXgene Blood RNA Kit IVD **(**QIAGEN, Hilden, Germany) according to the manufacturer's instructions. To ensure complete DNA removal, on-column DNase digestion was performed and RNA was eluted in 15–20 μl RNase-free water.

### RT-qPCR

Per sample 2 μg of RNA was reverse transcribed into cDNA using High-Capacity cDNA Reverse Transcription Kit (Applied Biosystems, Foster City, California, USA). Relative gene expression was determined using Power SYBR Green master Mix (Applied Biosystems) on a StepOnePlus real time PCR system (Applied Biosystems). The following cycling conditions were used: Hot-start activation (95°C, 2 min), denaturation for 40 cycles (95°C, 15 s) and annealing/extension (60°C, 60 s). Melting curve analysis was done to confirm amplification specificity. For each sample, qPCRs were run in triplicates. Cycle thresholds (Ct) were determined and relative mRNA expression to TATA-binding protein (TBP) (reference gene) was calculated using the ΔCt method. Primer sequences are listed in Table 1.

**Table 1:** Primer sequences used for RT-qPCR for furin, ACE2, and TATA-binding protein (TBP).

**Gene**	**Primer pair**	**Sequence (5^**′**^-3^**′**^)**	**Tm (^**°**^C)**
TBP	Fwd	GAATATAATCCCAAGCGGTTTG	56.5
	Rev	ACTTCACATCACAGCTCCCC	59.4
Furin	Fwd	GGCAAAGCGACGGACTAAAC	59.4
	Rev	CGTCCAGAATGGAGACCACA	59.4
ACE2	Fwd	TGTAAAACGACGGCCAGT	53.7
	Rev	CAGGAAACAGCTATGACC	53.7

### Statistical Analyses

All statistical analyses were done using GraphPad Prism version 8 (GraphPad software, California, USA). Data were tested for normality using the Shapiro-Wilk normality test and differences between two groups were assessed by *T*-test (paired or unpaired) as appropriate. For multiple comparisons, One-way ANOVA with Dunnett's multiple comparisons test, if normally distributed, or with Friedman test using Dunn's multiple comparisons test, if not normally distributed, were applied. Fisher exact test was applied for differences between study groups. The significance level for the analyses was set at a p-value of ≤ 0.05.

### Ethical Approvals

Dithranol study (Clinical Trials.gov no. NCT02752672) in psoriatic patients was completed in cooperation with the Department of Dermatology, Klagenfurt State Hospital, Austria. Clinical trial procedures of the dithranol study were approved by the ethics committee of the federal state of Carinthia, Austria (protocol number A23/15) and all participants gave written informed consent in accordance with the principles of the Declaration of Helsinki. The data collection in the patients of the Psoriasis Registry Austria was approved by the Ethical Committee of the Medical University of Graz, Austria (protocol number 21–094 ex 09/10) in accordance with the Declaration of Helsinki. All procedures and analyses of this study had additional approvement by the Ethical Committee of the Medical University of Graz (protocol number 32–587 ex 19/20).

## Results

**Figure 1:**
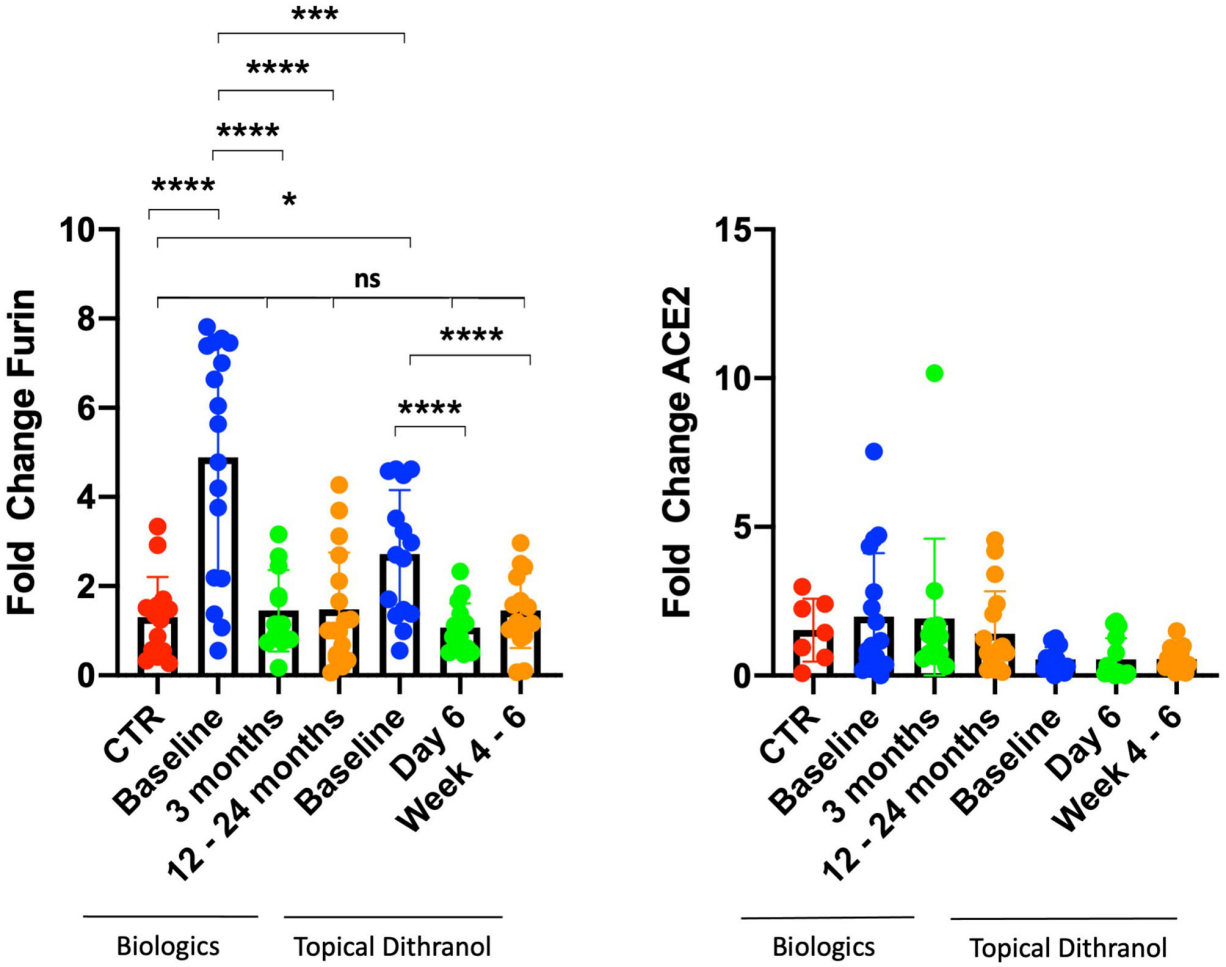
Furin and ACE2 expression levels in peripheral blood cells. Furin and ACE2 expression levels in peripheral blood cells from patients with psoriasis treated either with biologics (secukinumab, *n* = 12; ustekinumab, *n* = 5) or topical dithranol (*n* = 15) compared to healthy controls (CTR) (*n* = 12). *p < 0.05; ***p < 0.001; ****p < 0.0001; ns not significant.

This study analyzed in total blood samples from 12 healthy individuals (66.7% women) and 32 psoriasis patients, consisting of 8 (25.0%) women and 24 (75.0%) men from two separate studies. Seven patients (21.9%) were suffering from psoriatic arthritis (Table 2). There were a few differences between patients treated with dithranol and patients treated with biologics, who more frequently suffered from psoriatic arthritis (41.2 vs. 0%, *p* = 0.008) and concomitant hepatic steatosis (35.3 vs. 6.7% *p* = 0.024). Other characteristics such as age at study entry, BMI or PASI did not show any significant differences at start of the respective treatment. Healthy individuals had less comorbidities and were mostly of normal weight (Table 2). Furin mRNA expression levels at start of treatment were significantly elevated in the group treated with biologics by 4.9 (±2.6) fold (*p* < 0.0001) and in the dithranol group by 2.7 (±1.4) fold (*p* < 0.001) (Figure 1), compared to controls. Overall, no correlation for PASI and furin expression at baseline was observed (*p* = 0.868). Additionally, no differences in furin expression could be observed regarding gender (*p* = 0.236) or obesity (0.592) in a combined analysis (Table 3). However, there was a trend for arthritis patients to express more furin than patients with psoriatic skin involvement only (5.26 ± 2.30 vs. 3.48 ± 2.27, *p* = 0.078) (Table 3). PASI significantly decreased from 11.5 ± 4.5 at baseline to 2.0 ± 3.9 at month 3 and to 0.3 ± 0.5 after 12 to 24 months of treatment with biologics (*p* < 0.0001) (Table 2). For PASI improvement of patients treated with dithranol, please see our previously published work (45) and Table 2. Analyzing furin mRNA expression after treatment initiation with secukinumab or ustekinumab revealed a significant decrease in mRNA expression after 3 and 12 to 24 months, respectively (Figure 1). Similar findings were obtained for patients treated with dithranol, with significantly decreased expression levels 6 days after start of dithranol treatment and also at follow-up (4-6 weeks after dithranol treatment had been terminated).

**Table 2:** Characteristics of patients treated with dithranol or biologics.

**Demographics**			**Controls (*n* = 12)**	**Patients treated with dithranol (*n* = 15)**	**Patients treated with biologics (*n* = 17)**	***P*-value**
Patient characteristics	Mean age (SD) at study entry		36.7 (8.1)	42.9 (18.1)	35.4 (11.9)	0.274
	Number (%) of female patients		8 (66.7)	4 (26.7)	4 (23.5)	**0.037**
	Mean BMI (SD)		23.8 (5.2)	28.9 (7.7)	28.9 (9.0)	0.159
	Mean PASI (SD)	Baseline	NA	13.6 (10.6)	11.5 (4.5)	0.481
		Day 6 (dithranol)/month 3 (biologics)	NA	9.0 (6.3)	2.0 (3.9)	NA
		Week 4–6 (after dithranol)/month 12–24 (biologics)	NA	5.7 (6.7)	0.3 (0.5)	NA
Number (%) of common comorbidities	Arthritis (%)		0	0	7 (41.2)	**0.008**
	Depression		0	2 (13.3)	0	0.181
	Hypertension		0	4(26.7)	5 (29.4)	0.128
	Hyperlipidemia		0	0	3 (17.6)	0.102
	Hypothyreosis		0	2 (13.3)	0	0.181
	Hepatic steatosis		0	1 (6.7)	6 (35.3)	**0.024**
	Obesity		2 (16.7)	5 (33.3)	4 (23.5)	0.699

*Patient characteristics have been calculated using analysis of variances across all cohorts. Differences regarding comorbidities in the dithranol and biologic cohorts have been calculated with Fisher's exact test and student t test. Significant p-values are in bold. SD standard deviation, NA not applicable*.

**Table 3:** Furin expression at baseline in relation to certain patient characteristics.

**Characteristics**		**Fold change (SD)**	***P*-value**
Gender	men	4.16 (2.21)	0.236
	women	3.00 (2.74)	
Arthritis	yes	5.26 (2.30)	0.078
	no	3.48 (2.27)	
Obesity	yes	3.51 (2.38)	0.592
	no	4.01 (2.39)	

ACE2 expression levels did not differ from controls at any timepoint, regardless of biologic or topical treatment (Figure 1).

## Discussion

The treatment of psoriasis has become challenging with SARS-Cov2 raging across the world for almost a year and with no fast easing of this pandemia to be expected. However, analysis of virus characteristics together with retrospective patient cases has led to the identification of certain patient conditions endangering them to a more severe course of COVID-19 (13, 40). Cardiovascular and dysmetabolic conditions are also common among psoriasis patients, and a beneficial effect of anti-psoriatic therapy has already been suggested (46, 47). The proprotease furin, which is overexpressed in some conditions that are known as risk factors for severe COVID-19 infection, might play a major role in infection of host cells and virus replication. Accordingly, furin represents a promising target for medicamentous inhibition as well as biomarker, identifying high-risk patients (15, 16). So far furin has not been studied in psoriasis and most studies regarding COVID-19 and psoriasis referred to patients currently under systemic treatment. In this study, increased furin mRNA expression in peripheral blood in psoriasis patients was observed and a trend for an even higher expression in patients with concomitant arthritis discovered. We did not observe a correlation between PASI and furin mRNA expression levels, indicating that moderate to severe psoriasis *per se* increases expression of furin, irrespective of its severity. Furthermore, systemic biologic and topical dithranol treatment significantly reduced expression of furin, ultimately normalizing expression levels compared with healthy controls after 3 and 12 to 24 months upon treatment with biologics. Strikingly, furin levels quickly normalized after initiation of dithranol treatment, although PASI had only been reduced by 32.9% (from 13.6 to 9.0) at day 6 (Figure 1). Furthermore, it appears that dithranol treatment can sustainably normalize furin expression in patients with moderate to severe psoriasis, provided the disease remains improved as observed by PASI reduction of 58.1% (from 13.6 to 5.7) 4–6 weeks after end of treatment (Figure 1). It remains to be determined, if topical treatment including steroids and vitamin D can also reduce expression of furin in patients‘ blood. Moreover, it remains to be elucidated how exactly biologic or dithranol treatment lower furin levels in psoriasis patients.

Our findings may contribute to a better understanding of relatively low hospitalization rates in rheumatological patients treated with systemic drugs (although mainly with TNF-α inhibitors), who fell ill to COVID-19 and possibly explain why no significant increase in mortality or hospitalization was found for psoriasis patients under systemic treatment. A beneficial effect of biologic therapies on preventing cytokine storms and severe courses of COVID-19 has also been hypothesized by dermatologists recently (11). As furin has been found upregulated in leukocytes of rheumatological and psoriatic patients, these conditions may increase the risk for severe COVID-19 disease (15, 16, 25, 28). However, the treatment-induced normalization of furin expression observed in patients with psoriasis and psoriatic arthritis, could be important for risk management in those patients. Notably, similar risks for contracting COVID-19 or having a more severe course of disease have been observed in patients treated with biologics or topical therapies (48), possibly resulting from furin normalization in patients treated with biologics or topical therapy. Furthermore, the direct inhibition of TNF-α and an assumable normalization of furin expression in leukocytes could explain a better outcome in rheumatological patients treated with TNF-α inhibitors, as high levels of TNF-α are associated with a severe course of disease (38). However, one can only assume that TNF-α inhibitors reduce furin expression in a similar way to secukinumab, ustekinumab, and dithranol, as we could not study patients treated with TNF-α inhibitors. Therefore, further studies may investigate furin expression in psoriasis and rheumatological patients prior to and after treatment start with TNF-α inhibitors.

Notably, the normalization of furin expression in leukocytes could be important with regard to worse outcome in patients developing lymphopenia in COVID-19. However, as for rheumatological diseases (such as rheumatoid arthritis, Sjögren syndrome or systemic lupus erythematodes), it remains still unclear, if psoriasis *per se* may increase the risk for SARS-CoV2 infection, irrespective of disease severity and course.

Furthermore, ACE2 mRNA expression levels were not elevated at treatment start compared to healthy controls and did not change during topical or systemic therapy. However, recent studies revealed that ACE2 is hardly expressed in peripheral blood leukocytes, and little is known about factors altering its expression levels in blood leukocytes (49–52).

## Limitations

The small population size and differences in baseline characteristics (including a disbalanced gender ratio, BMI and comorbidities) between the cohorts and controls is a limitation to the study and may have contributed to the relatively higher furin expression levels at baseline in psoriasis patients. While increased furin expression has been reported in blood monocytes from obese patients (including patients with diabetes and atherosclerosis) (53), we could not detect significant differences between obese and non-obese patients of our in average relatively young study cohorts. Furthermore, no patient of this study had diabetes or atherosclerosis, conditions commonly associated with furin overexpression. Furin plays a crucial role in liver cancer (54), but its role in fatty-liver disease is unknown. Furthermore, we did not investigate furin mRNA expression in the skin or protein levels in plasma.

## Conclusion

Taken together, we may have been worried about the wrong psoriasis patients, as furin is significantly overexpressed in untreated patients, and, thus, these patients may be at risk for infection and a severe course of COVID-19. Our data indicate that the successful therapeutic treatment of psoriasis, irrespective of systemic biologic or topical dithranol treatment, can effectively reduce furin expression in blood leukocytes. Considering the ongoing discussion of a potential risk of elevated furin levels for a severe course of COVID infections, the successful treatment of psoriasis appears to limit patients risk for a severe COVID-19 course.

## Data Availability Statement

The raw data supporting the conclusions of this article will be made available by the authors, without undue reservation.

## Ethics Statement

The studies involving human participants were reviewed and approved by Ethics committee of the Medical University of Graz. The patients/participants provided their written informed consent to participate in this study.

## Author Contributions

TG, NG-S, and PW: had full access to all of the data in the study, take responsibility for the integrity of the data, the accuracy of the data analysis, concept, design, statistical analysis, and drafting of the manuscript. PW: supervision and obtained funding. TB, WW, WS, CP, and PW: administrative, technical, or material support. All authors critical revision of the manuscript for important intellectual content, acquisition, analysis, or interpretation of data.

## Conflict of Interest

PW has received research grants, speaker and/or consulting honoraria, and/or travel refunds from AbbVie, Amgen GmbH, Almirall, Celgene, Eli Lilly, Janssen, Leo Pharma, Novartis, Merck Sharp & Dohme, Sandoz and Pfizer. TG has received a travel grant from Novartis. WW has received speaker and/or consulting honoraria and/or travel refunds from AbbVie, Amgen GmbH Almirall, Celgene, Eli Lilly, Janssen, Leo Pharma, Novartis, Merck Sharp & Dohme, Sandoz and Pfizer. The remaining authors declare that the research was conducted in the absence of any commercial or financial relationships that could be construed as a potential conflict of interest.
